# Phenotypic subtyping via contrastive learning

**DOI:** 10.1101/2023.01.05.522921

**Published:** 2023-01-06

**Authors:** Aditya Gorla, Sriram Sankararaman, Esteban Burchard, Jonathan Flint, Noah Zaitlen, Elior Rahmani

**Affiliations:** 1 Bioinformatics Interdepartmental Program, University of California, Los Angeles, Los Angeles, CA, USA; 2 Department of Computer Science, University of California, Los Angeles, Los Angeles, CA, USA; 3 Department of Computational Medicine, David Geffen School of Medicine, University of California, Los Angeles, Los Angeles, CA, USA; 4 Department of Human Genetics, University of California, Los Angeles, Los Angeles, CA, USA; 5 Department of Medicine, University of California, San Francisco, San Francisco, CA, USA; 6 Department of Bioengineering and Therapeutic Sciences, University of California, San Francisco, San Francisco, CA, USA; 7 Department of Psychiatry and Behavioral Sciences, Brain Research Institute, University of California, Los Angeles, Los Angeles, CA, USA; 8 Department of Neurology, David Geffen School of Medicine, University of California, Los Angeles, Los Angeles, CA, USA

## Abstract

Defining and accounting for subphenotypic structure has the potential to increase statistical power and provide a deeper understanding of the heterogeneity in the molecular basis of complex disease. Existing phenotype subtyping methods primarily rely on clinically observed heterogeneity or metadata clustering. However, they generally tend to capture the dominant sources of variation in the data, which often originate from variation that is not descriptive of the mechanistic heterogeneity of the phenotype of interest; in fact, such dominant sources of variation, such as population structure or technical variation, are, in general, expected to be independent of subphenotypic structure. We instead aim to find a subspace with signal that is unique to a group of samples for which we believe that subphenotypic variation exists (e.g., cases of a disease). To that end, we introduce Phenotype Aware Components Analysis (PACA), a contrastive learning approach leveraging canonical correlation analysis to robustly capture weak sources of subphenotypic variation. In the context of disease, PACA learns a gradient of variation unique to cases in a given dataset, while leveraging control samples for accounting for variation and imbalances of biological and technical confounders between cases and controls. We evaluated PACA using an extensive simulation study, as well as on various subtyping tasks using genotypes, transcriptomics, and DNA methylation data. Our results provide multiple strong evidence that PACA allows us to robustly capture weak unknown variation of interest while being calibrated and well-powered, far superseding the performance of alternative methods. This renders PACA as a state-of-the-art tool for defining *de novo* subtypes that are more likely to reflect molecular heterogeneity, especially in challenging cases where the phenotypic heterogeneity may be masked by a myriad of strong unrelated effects in the data.

## 1 Introduction

Heterogeneity in the underlying biological mechanisms of complex disease and traits has practical and clinical implications: undifferentiated cases of a disease may represent the action of a variety of underlying causal processes, each of which may have a different prognosis or respond to a different treatment. One classic example is the differentiation of diabetes mellitus into type 1 (insulin responsive) and type 2 (non-responsive) [1]. Other examples include the sub-classification of autoimmune thyroid disease into Hashimoto thyroiditis and Graves’ disease, the most common causes of hypothyroidism and hyperthyroidism, respectively [2], and the stratification of breast cancer patients based on estrogen receptor (ER) proteins on the surfaces of tumor cells (ER+/−), which is indicative of prognosis and response rates for drugs that interact with ER [3].

The term “phenotypic heterogeneity” is often used to describe observed variation of a given phenotype between individuals, irrespective of whether heterogeneity in the molecular background of the phenotype contributes to that variation. Here, we consider phenotypic heterogeneity in the sense of variability in the molecular basis of a phenotype. Such heterogeneity reflects possible variation or difference in the mechanism of the phenotype across individuals, which may be driven by genetics [4–10], environmental exposures and/or by the interaction thereof [10–18]. In fact, phenotypic heterogeneity is suspected yet unknown or not fully characterized for many conditions, including neurodegenerative diseases (e.g., Alzheimer’s disease [19] and dementia [20]), autoimmune diseases (e.g., rheumatoid arthritis and systemic lupus erythematosus [21]), and psychiatric disorders (e.g., major depressive disorder (MDD) [22, 23] and post-traumatic stress disorder [24]).

In the absence of the right subgrouping, phenotypic heterogeneity may compromise statistical analysis by leading to a substantial power loss and potentially low reproducibility rates in detecting and understanding the underlying mechanisms of heterogeneous phenotypes [10, 25–27]. Since the sub-classification or heterogeneity nature of the molecular background of phenotypes is typically unknown, it becomes a computational and statistical challenge to find surrogates to the subtypes.

Numerous studies have defined phenotypic subtypes based on histology [28, 29], genomics [30–32], electronic health records [33], or other phenotypically- or clinically-determined scores (e.g., [23, 34–36]). This approach occasionally reveals disease subgrouping that facilitates the detection of novel genetic signals. For example, using clinical and familial criteria, subgroups have often been proposed for lithium responsive bipolar disorder [34] and mood incongruent psychosis [35]; association studies with MDD indicate that its underlying genetic basis differs between those who have and those who have not experienced adversity [23]; and subtype characterization using gene expression signatures identified classes of tumors with distinct patterns of chromosomal alterations [37]. However, heterogeneity in the molecular basis of disease might not be observed phenotypically (e.g., both type 1 and type 2 diabetes share some similar symptoms), and in general, ad hoc clinically- or phenotypically-derived subgrouping is not guaranteed to capture variation in the underlying mechanisms.

Arguably, the most common systematic approach for defining *de novo* subtypes in a data-driven fashion with no prior information is data clustering and dimensionality reduction. This subtyping approach employs methods ranging from classical clustering and dimensionality reduction methods, such as K-means [31], hierarchical clustering [38, 39], and principal components analysis (PCA) [40–42], to more modern deep-learning based methods, including various types of autoencoders and other generative network models (see [43] for a review). These methods can possibly be used in combination with feature selection or feature weighting based on domain-specific prior knowledge [44, 45]. Importantly, all of these methods are essentially geared towards learning latent representation of samples based on the dominant sources of variation in a given dataset. This renders such methods ineffective in the typical case where phenotypic heterogeneity is not mirrored by the top axes of variation in the data. Particularly, the most dominant sources of variation in biological data most often reflect various population- or sample-related factors that can be independent of causal phenotypic variation, such as population structure [46, 47], cell-type composition [48], and technical variation [49, 50].

**Figure 1:**
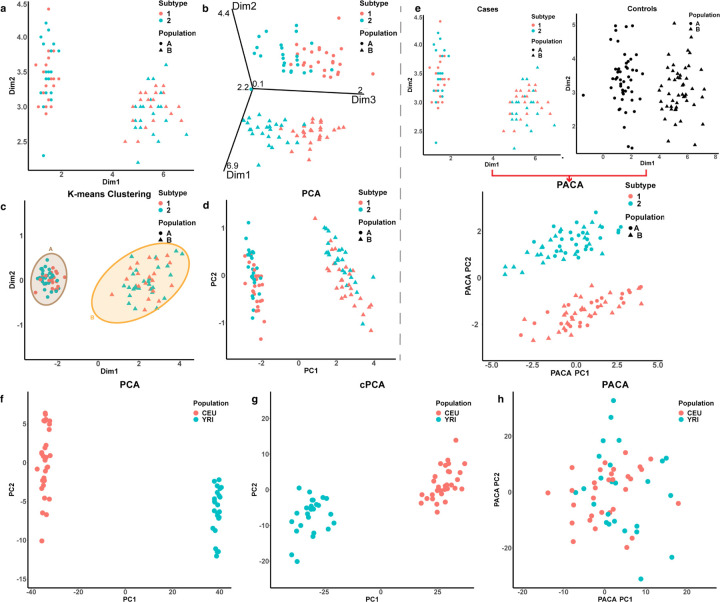
An illustration of learning weak sub-phenotypic signals. (a) Dimensions 1 and 2 of the data stratify samples by population (circles/triangles) rather than by subtype (red/green). (b) A third dimension of the data, which reflects a weaker signal, stratifies observations into the two subtypes. (c-d) A standard K-means clustering approach and dimensionality reduction using PCA surface population structure, the dominant source of variation in the data. (e) Contrasting cases with controls (which include no subtype signal) allows PACA to isolate cases-specific variation (i.e., subtypes), regardless of the rank of the signal. (f)-(h) Evaluation of the top two components of PCA, cPCA, and PACA under a scenario with no subphenotypic heterogeneity, in which samples from the HapMap data [51] were randomly assigned with a case/control status (58 YRI and 58 CEU individuals; based on a randomly selected set of 10,000 SNPs). Two outlier samples were excluded for visualization purposes).

In some cases, the most dominant sources of variation in a dataset can lead to meaningful subtyping – perhaps most notably in genomic data from tumors, which include strong structure induced by the large number of mutations and genomic changes with cancer [52, 53]. However, the molecular signals underlying complex traits are most often expected to be weak (i.e., small effect sizes) and sparse; that is, the vast majority of the features available in a given dataset are not causal or statistically correlated with the phenotype of interest [54, 55]. Adjusting or conditioning the analysis on factors (i.e., as covariates) that are not expected to be statistically related to the subphenotypic signal can in principle alleviate this problem. Yet, in practice, unknown factors with unknown effects typically exist, and some of those factors may obscure the subphenotypic signal unless accounted for in the analysis. Therefore, whether conditioning on known covariates or not, a standard approach that relies on dominant variation in the data, in general, is not expected to allow us to robustly target phenotypic heterogeneity (or, more generally, a particular unknown weak variation of interest; Figure 1a–d).

Given the potential impact of identifying phenotypic heterogeneity on our ability to improve healthcare outcomes, an important question is thus whether we can systematically learn *de novo* phenotypic subtypes in cases where the phenotypic heterogeneity constitute only a small portion of the variation in the data and no prior information on the nature of the phenotypic heterogeneity is known.

### Related work

Targeting weak sources of variation in a dataset can in principle be achieved by conditioning on background data and introducing subspace-specific regularization [56]. More recently, a method following this approach, contrastive PCA (cPCA) [57], has been proposed and applied for the task of subtyping and sample subgrouping using protein and gene expression data. cPCA seeks to learn directions of variation that are enriched in a target dataset (e.g., cases) compared a background dataset (e.g., controls), which reflects a contrastive learning paradigm by which a weak form of supervision (target versus background labels) informs the unsupervised learning of patterns in the target data. In theory, the contrastive learning approach allows us to study subphenotypic variation in a group of cases by accounting for all variation that exists in controls, thus, dismissing the need for explicitly considering the (unknown) right set of covariates if taking a more straightforward approach of analyzing the cases alone.

The cPCA algorithm was demonstrated to successfully capture subgrouping information on several tasks [57], however, it is expected to be limited in scenarios of unsupervised detection of *de novo* subtypes where we do not have a prior knowledge about the right level of subspace regularization. Concretely, cPCA indicates for the user several possible levels of subspace regularization based on similarity of their induced subspaces in terms of their principle angles, yet, this may lead to tagging confounders. In the absence of a priori knowledge of subtypes, the analyst is likely to misspecify the regularization parameter, which may lead to falsely tagging arbitrary patterns in the target data as subphenotypic variation (Figure 1g). Critically, since biological confounders tend to replicate across independent datasets from the same population, tuning the regularization parameter via standard cross validation procedures is in general not possible.

Finally, deep learning methods for contrastive learning exist (e.g., contrastive variational autoencoders [58]), however, they require large sample sizes in order to learn models with a large number of parameters and address the sensitivity of deep learning to architecture and hyperparameter tuning. Given that large sample sizes are often not available in biological datasets, we do not consider such methods in our evaluation.

### Contributions

We propose Phenotype Aware Components Analysis (PACA), a method for robustly capturing weak variation of interest in high-dimensional data. PACA is a contrastive learning algorithm leveraging Canonical Correlation Analysis (CCA) to learn patterns in a given target dataset that cannot be found in background data. Given case-control data of any modality, PACA highlights the cases-specific dominant variation of the subspace that is not affected by control variation as a putative representation of phenotypic heterogeneity. By defining background variation as any biological or technical variation that is shared between cases and controls and is stronger than the cases-specific variation in the data, we cast PACA as an estimation algorithm, which subverts the need for a vague and hard-to-tune contrastive hyperparameter.

## 2 Methods

### 2.1 Preliminaries

#### Problem setup

We consider a low-rank model for high-dimensional data coming from a target population and a background population. Both populations share the same biological and non-biological sources of variation, with the exception of an additional source of variation that is unique to the target population and represents subphenotypic signal. Hereafter, we refer to the target and background groups as “cases” and “controls”, as a representation of the status of the samples in the data with respect to a condition of interest.

Let $X\in {\mathbb{R}}^{m\times n_{1}}$ be a matrix of measurements of *m* features for *n*_1_ cases, and let $Y\in {\mathbb{R}}^{m\times n_{0}}$ be a matrix of measurements of the same *m* features for *n*_0_ control samples, such that *m* > max(*n*_0_, *n*_1_). We consider the following descriptive model:

(1) $$X=W^{0}Z_{X}^{0}+W^{1}Z_{X}^{1}+E_{X},\;{\left(E_{X}\right)}_{i}∼N\left(0,\sigma^{2}I_{m}\right)$$

(2) $$Y=W^{0}Z_{Y}^{0}+E_{Y},\;{\left(E_{Y}\right)}_{i}∼N\left(0,\sigma^{2}I_{m}\right)$$

$W^{0}\in {\mathbb{R}}^{m\times k_{0}}$, $Z_{X}^{0}\in {\mathbb{R}}^{k_{0}\times n_{1}}$, $Z_{Y}^{0}\in {\mathbb{R}}^{k_{0}\times n_{0}}$ represent the directions (in the features space ${\mathbb{R}}^{m}$) of a *k*_0_-dimensional low-rank signal (typically *k*_0_ << min(*n*_0_, *n*_1_)) and sample-specific structures for the samples in *X*, *Y*, respectively, by which the shared sources of variation across cases and controls are encoded. $W^{1}\in {\mathbb{R}}^{m\times k_{1}}$, $Z_{X}^{1}\in {\mathbb{R}}^{k_{1}\times n_{1}}$ represent the directions of a *k*_1_-dimensional low-rank signal and sample-specific structure for the samples in *X*, respectively, by which the cases-specific sources of variation are encoded.

Since $Z_{X}^{1}$ represents variation that cannot be attributed to any source of variation that exists in control samples it can be interpreted as variation due to heterogeneity among cases. In our context, we refer to this cases-specific variation as phenotypic heterogeneity or phenotypic subtypes; of note, while the latter may imply a categorical classification of samples into a constant number of groups, the former conceptually allows a more flexible characterization of the cases-specific variation by considering a spectrum to represent the phenotypic heterogeneity. Our goal is to learn $Z_{X}^{1}$ up to a linear transformation. We are particularly interested in a regime in which the cases-specific signals are weaker than (at least some of) the sources of variation that are shared across cases and controls (i.e., *W*^1^ spans less variation compared to *W*^0^).

#### Orthogonality assumption

If *W*_1_ is in the subspace spanned by *W*_0_ then all the structure in *X* and *Y* is spanned by the same subspace. This case corresponds to a scenario in which there is no cases-specific variability in the data, and although subphenotypic variation may exist, $Z_{X}^{1}$ appears non-identifiable without additional supervision. Here, we make the assumption that $W^{0}⊥W^{1}$. That is, we assume that the axes of common variation across cases and controls are orthogonal to the cases-specific variation. This assumption suggests that under the model in Eq. (1)–(2) a sensible approach for learning the cases-specific variation is to find sources of variation in *X* that do not exist in *Y*. Neglecting this assumption, while not allowing further supervision, may lead to under-correction for shared sources of variation across cases and controls. This, in turn, can lead to falsely tagging background variation as phenotypic heterogeneity (Figure 1f–h).

Notably, linear effects with the condition under study, in general, do not describe a dichotomous relation between features and the condition but rather a statistical one. Therefore, features with such linear effects are typically expected to vary in control samples too. Yet, this for itself does not nullify the orthogonality assumption, which does not concern feature-specific variation but rather reflects the assumption that control samples do not exhibit a systematic variation that is unique to cases. That being said, in practice, the orthogonality assumption may be violated since phenotypic heterogeneity may also be correlated with factors that exist in controls, such as population structure and demographics. As we later show empirically, violation of the orthogonality assumption will lead to a decrease in sensitivity to capture cases-specific variation. However, working under this assumption has the benefit of avoiding under-correction for the shared variation across cases and controls.

#### Contrastive PCA is limited under the orthogonality assumption

Using the same notations as above, given a parameter *α* > 0, cPCA finds the top contrastive principal component (PC) by solving [57]:

(3) $$v^{*}=\underset{v\in {\mathbb{R}}^{m}}{\text{argmax}}\;v^{⊤}XX^{⊤}v-\alpha v^{⊤}YY^{⊤}v\;\text{ s.t., }∥v{∥}_{2}=1$$

In words, cPCA aims at finding the dominant axis of variation in *X* while controlling at a level associated with *α* for the variation this axis describes in *Y*. This framework allows us in principle to learn the cases-specific variation $Z_{X}^{1}$ described in Eq. (1)–(2). Particularly, setting *α* = ∞ allows cPCA to incorporate the orthogonality assumption and account for all shared sources of variation across *X* and *Y*. A solution to Eq (3) while setting *α* = ∞ requires that $\left\|Y^{⊤}v^{*}\right\|=0$ in order to avoid a negative infinity value for the objective. Therefore, v* must be orthogonal to the subspace spanned by the columns of *Y*, i.e., $v^{*}\in \text{null}(Y):=\left\{v|Y^{⊤}v=0_{m}\right\}$, where 0_*m*_ is an *m*-length vector of zeros. Effectively, since *X*, *Y* are full-rank matrices due to the i.i.d. components of variation in Eq. (1)–(2), v* must be orthogonal to an *n*_0_-dimensional subspace defined by to all samples in *Y*.

If *n*_0_ ≥ *m* then v* must be orthogonal to the entire *m*-dimensional features space, hence we get v* = 0_*m*_ irrespective of the rank of *X*. Here, we are interested in the case where max(*n*_0_, *n*_1_) < *m*, therefore v* is non-trivial, however, following the rank–nullity theorem, v* is restricted to an *m*−*n*_0_ dimensional subspace of ${\mathbb{R}}^{m}$. Clearly, as *n*_0_/*m* approaches 1, cPCA will not allow us to capture the direction that spans the subphenotypic signals. Intuitively, this approach is suboptimal since it conditions on the entire n_0_-dimensional subspace in *Y* rather than on the lower-dimensional subspace that captures only the low-rank signals in the data. This suggests that a better approach is to condition only on sources of variation that exist in both *X*, *Y*, which are expected to be of lower dimension compared with the dimension of the observed data. This is exactly the key idea behind PACA.

### 2.2 PACA: Phenotype-Aware Component Analysis

#### Capturing subphenotypic variation using PACA

Using a singular value decomposition we can consider an alternative formulation for the model in (1)-(2):

(4) $$X=U^{0}{\text{Σ}}_{X}^{0}{\left(V_{X}^{0}\right)}^{⊤}+U^{1}{\text{Σ}}_{X}^{1}{\left(V_{X}^{1}\right)}^{⊤}+E_{X},\;U^{0}⊥U^{1}$$

(5) $$Y=U^{0}{\text{Σ}}_{Y}^{0}{\left(V_{Y}^{0}\right)}^{⊤}+E_{Y}$$

where each of $U^{0}\in {\mathbb{R}}^{m\times k_{0}}$, $V_{X}^{0}\in {\mathbb{R}}^{n_{1}\times k_{0}}$, $V_{Y}^{0}\in {\mathbb{R}}^{n_{0}\times k_{0}}$, $U^{1}\in {\mathbb{R}}^{m\times k_{1}}$, and $V_{X}^{1}\in {\mathbb{R}}^{n_{1}\times k_{0}}$ forms an orthonormal basis. Under this presentation, we are interested in learning $V_{X}^{1}$, a linearly-transformed surrogate for $Z_{X}^{1}$ in Eq. (1). Given the directions of the shared sources of variation *U*^0^, note that

(6) $$E\left[U^{0}{\left(U^{0}\right)}^{⊤}X\right]=U^{0}{\text{Σ}}_{X}^{0}{\left(V_{X}^{0}\right)}^{⊤}$$

which established a way to remove the expected effect it induces on *X* (i.e., the signals in *X* that are coming from sources of variation that exist in controls). Our PACA algorithm therefore estimates and removes the effects of *U*^0^ on *X*, followed by PCA for capturing $V_{X}^{1}$. Our main focus is thus estimating *U*^0^.

Since *U*^0^ is found in both *X*, *Y*, we can estimate it by employing a canonical correlation analysis (CCA) [59]. Importantly, unlike in a typical application of CCA, where we wish to find linear transformations of the features that yield the highest correlation between the samples in two datasets (i.e., vectors in the samples space), here, we seek linear transformations of the samples that provide vectors in the features space ${\mathbb{R}}^{m}$. Specifically, we find the first pair of canonical variables by solving:

(7) $$\hat{a},\hat{b}=\underset{a\in {\mathbb{R}}^{n_{1}},b\in {\mathbb{R}}^{n_{0}}}{\text{argmax}}a^{⊤}X^{⊤}Yb,\;\text{ s.t. }∥Xa{∥}_{2}=1,∥Yb{∥}_{2}=1$$

Setting ${\hat{u}}_{1}^{0}=X\hat{a}$ yields the representation in *X* of the strongest direction of shared variation across *X* and *Y*. Let *S*_*XY*_ be the empirical sample cross-covariance of the matrices *X* and *Y*, the solution for $\hat{a}$, $\hat{b}$ is known to be given by the eigenvector that corresponds to the top eigenvalue of $S_{XX}^{-1}S_{XY}S_{YY}^{-1}S_{Y\;X}$ and the eigenvector that corresponds to the top eigenvalue of $S_{YY}^{-1}S_{Y\;X}S_{XX}^{-1}S_{XY}$, respectively [59]. This procedure can be repeated iteratively by restricting the vectors ${\hat{u}}_{r}^{0}$ to be orthogonal to the previous canonical variables ${\hat{u}}_{1}^{0},\ldots ,{\hat{u}}_{r-1}^{0}$ (and similarly for the variables of *Y*). Eventually, the collective of these vectors ${\hat{U}}^{0}$ can be used for removing the shared sources of variation from *X* following Eq (6); see a summary of PACA in Algorithm 1. As we discuss next, choosing the number of components *r* to be used can be informed by the structure induced in *X* and *Y* by the shared variation.


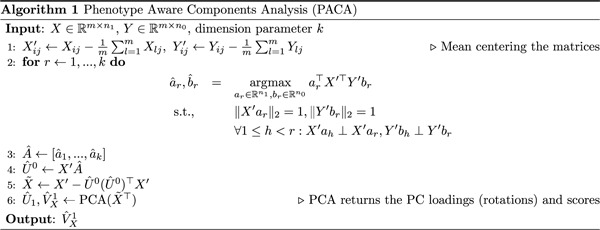


We note that the CCA step in PACA imposes the limitation *m* ≥ max(*n*_0_, *n*_1_). In practice, there may be datasets in which this condition does not hold. In such cases, we can in principle apply PACA on a subset of the samples, however, this is clearly sub-optimal to exclude data. Instead, we can use a randomized version of PACA that can operate under the setup *m* < max(*n*_0_, *n*_1_) by using multiple random subsets of the data for estimating the full sample covariance matrix of *X* (while accounting for the shared sources of variation between *X* and *Y*). See Supplementary Methods and Supplementary Algorithm 2 for details.

#### Estimating the dimension of the shared variation

In typical settings, the parameter *k*_0_ of the dimension of *U*^0^ is unknown. We may estimate it by setting an estimate ${\hat{k}}_{0}$ to be the largest value *r* that reveals a significant correlation between the correlated subspace of *X* and *Y* induced by ${\hat{a}}_{r}$, ${\hat{b}}_{r}$; this can be achieved, for example using Bartlett’s Chi-squared test or Rao’s approximate F statistic [60]. However, in practice, we found that in the data we analyzed *k*_0_ seems to be very large (typically a few hundreds). Hence, this approach can lead to over-regularization and reduction in statistical power due to the unnecessary removal of a large number of axes of shared variation. In order to see why, recall that our goal is to be able to systematically target variation of interest that is typically weaker than the dominant sources of variation in the data. While standard dimensionality reduction tools cannot achieve this when applied to the original data, we expected them to be effective in revealing the target variation if applied to a residualized (adjusted) version of the data in which all sources of variation that are stronger than the target variation are removed. In other words, there is no need to estimate the true *k*_0_ and remove a structure of this dimension from the data prior to applying dimensionality reduction. Instead, we wish to find what is the minimal number of axes of variation *k* that we need to remove in order to reveal variation that is unique to cases. Below, we provide a brief description of the algorithm; see Algorithm 2 for complete details.

Given all shared axes of variation learned from the data using the procedure in Eq. (7), we learn *k* ∈ {1, …, min(*n*_0_, *n*_1_) using binary search as follows. At each given candidate *k*, we evaluate the variance of the top PC we calculate from the residualized *X* accounting for the top *k* shared axes of variation; this same PC, which may reflect cases-specific variation, is also used for evaluating the variance it can explain in the residualized *Y* matrix. Comparing these variances to ”null” variances obtained while permuting the loadings of the PC allows us to call whether accounting for *k* axes of shared direction is sufficient to detect structure that is unique to *X*. This evaluation can lead to one of four scenarios, based on which we decide on the next partition of the binary search: there can be (i) significant variation in cases but not in controls, (ii) significant variation in cases and controls, (iii) no variation in cases or controls, or (iv) significant variation in controls but not in cases.

Scenarios (i) and (iii) lead us to consider lower values of *k* due to possible over correction. The former means we revealed cases-specific variation, yet, we may be able to refine the signal if we can identify it using lower *k*, and the latter indicates that all the structure in the data has been removed. Scenario (iv) indicates a violation of our model assumption, which leads to termination, and lastly, scenario (ii) indicates residual shared variation, which suggests increasing *k*. Empirically, we found that considering a threshold *γ* on the maximum ratio between the variance of *X*’s PC and the variance it explain in *Y* under scenario (ii) is more stable and improves performance. Specifically, if the ratio between the variances of *X*, *Y* is greater than a predefined threshold then we handle this case similarly to case (i), and otherwise, we assume under-correction and consider a larger *k*. Throughout our analysis, unless stated otherwise, we used *γ* = 10.

### 2.3 Evaluation metrics and Datasets

See Supplementary Methods for complete details about datasets, data simulation, evaluation metrics, as well as complete technical details about the data analysis and implementation of PACA.


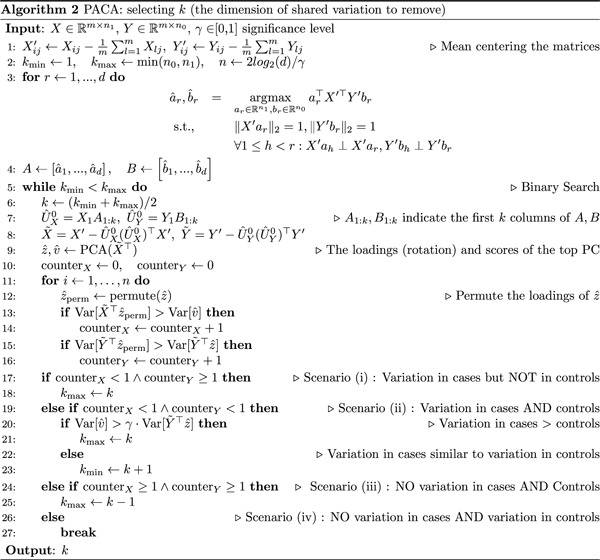


## 3 Results

### 3.1 PACA is calibrated under the null

A desired property of a method for capturing phenotypic heterogeneity is being calibrated under the null: given data with no phenotypic heterogeneity (i.e., the null case) and a prespecified confidence level. The method should be able to determine that no subphenotypic variation exists while controlling for the target type I error rate that corresponds to the prespecified confidence level. In order to verify that PACA is calibrated under the null, we applied it to data with no phenotypic heterogeneity by randomly sampling two groups of healthy individuals we labeled as “cases” and “controls” from each of the UK Biobank and the PsychENCODE datasets [61, 62](Supplementary Methods). We compared PACA with cPCA [57] and standard PCA, and using a permutation testing scheme we evaluated whether the structure found by each method represents phenotypic variation (Supplementary Methods). Empirically, we found that requiring cPCA to automatically select a single regularization parameter across a range of possible default values always favors no regularization (i.e., equivalent to PCA). We therefore set cPCA with a regularization parameter *α* = ∞, which we expected will favor the performance of cPCA in the calibration analysis. We found the PACA is calibrated and admits the lowest error rates among the different methods; for example, in the experiment using the PsychENCODE data PACA is calibrated with <5% error at a target type I error rate of 5%, yet cPCA and PCA demonstrate approximately 7% and 13% type I error, respectively (Supplementary Figure S1).

### 3.2 PACA is well-powered to capture phenotypic subtypes

We next evaluated PACA under the scenario where phenotypic heterogeneity does exist. To that end, we simulated high-dimensional data representing cases and controls. Specifically, every sample was generated by combining sample-specific structure with axes of variation that were shared across all samples. Then, we randomly split cases into two subtypes and added an additional weak and sparse cases-specific signal that differentiate between the two subtypes (Supplementary Methods).

In order to establish baseline performance we also considered standard PCA and cPCA. Since the latter depends on a regularization hyperparameter *α*, which is generally unknown, we considered two approaches. First, we applied cPCA while setting *α* = ∞, which allowed us to guarantee that cPCA learns variation that is strictly unique to cases. Second, we allowed cPCA to automatically recommend ten values of *α* based on the authors’ suggested algorithm for identifying subspaces that are maximally distinct from each other based the principle angles [57]. Then, we set the final *α* to the value that resulted in the best performance. This procedure represents a hypothetical scenario in which the subtype information is known and can be used for selecting the best *α*. This evaluation therefore reflects an over-optimistic evaluation of the performance of cPCA in practice. As before, setting cPCA to automatically select a single value of *α* always resulted in no regularization (i.e., *α* = 0, which is equivalent to a standard PCA).

**Figure 2:**
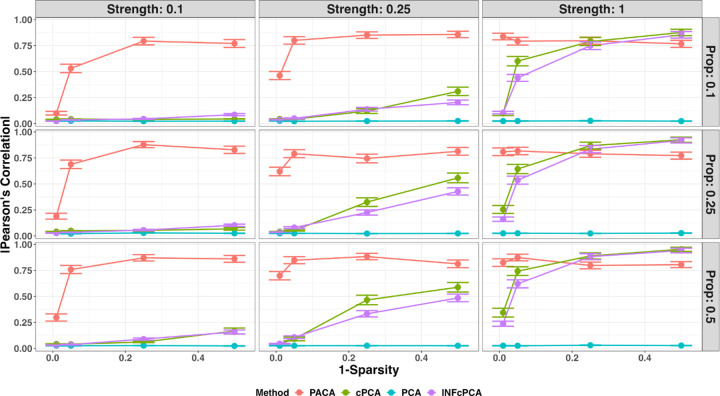
Evaluation of power to capture subphenotypic signal under simulations in the presence of two subtypes (*n* = 2000, *m* = 2000, *k*_0_ = 400). Performance was evaluated for PCA, PACA, cPCA with the best performing regularization parameter *α* among a range of values suggested by cPCA, and cPCA while setting the regularization parameter to *α* = ∞ (INFcPCA). Presented is the linear correlation of the top component of each method with the simulated subtype as a function of the signal sparsity (lower values mean more sparse), across a range of signal strengths (columns) and levels of imbalance (Prop) between the prevalence of the two subtypes (rows). The performance of every combination of parameters was averaged across 100 simulated datasets and vertical bars represent standard errors.

Our results show that except for the case of very strong signals PACA outperforms cPCA in all settings, in spite of our over-optimistic evaluation of cPCA (Figure 2). Particularly, we observe that unlike cPCA, PACA is well-powered even in cases of very sparse signals (Figure 2). We further examined the performance of the different methods under a regime where the orthogonality assumption is violated and the subtype information is correlated with the shared sources of variation between cases and controls (Supplementary Methods). As expected, we notice a substantial drop in power for all methods when the orthogonality assumption is violated (Supplementary Figure S2). Notably, PACA appears to be the most robust method under this regime in cases of weak to moderate correlation between the subtype classification and the shared sources of variation (Supplementary Figure S2b). In cases of severe violation of the orthogonality assumption, the power of all methods drop considerably, effectively not allowing to detect subtypes (Supplementary Figure S2c,d)

Finally, in order to gain more insight into the superior performance of PACA compared with cPCA, we compared the simulated dimension of shared variation between cases and controls (*k*_0_) to the dimension of shared variation that was removed by PACA. We found that in cases of weak subphenotypic signals PACA tends to adjust the data for a number of axes of shared variation that approximately matches the true simulated dimension of shared variation (Supplementary Figure S3). As the strength of the subphenotypic signal increases, a larger subspace of the space that defines the shared sources of variation is expected to include signals that are weaker than the subphenotypic signal. PACA leverages this insight and estimates the “effective” (i.e., minimal) dimension of shared variation that needs to be removed in order to detect subphenotypic signal (i.e., rather than removing all the shared variation; Algorithm 2); indeed, our analysis confirms that PACA adjusts for less axes of shared variation as the signal strength and density increase (Supplementary Figure S3).

### 3.3 Stratification of DNA methylation samples from admixed populations

**Figure 3:**
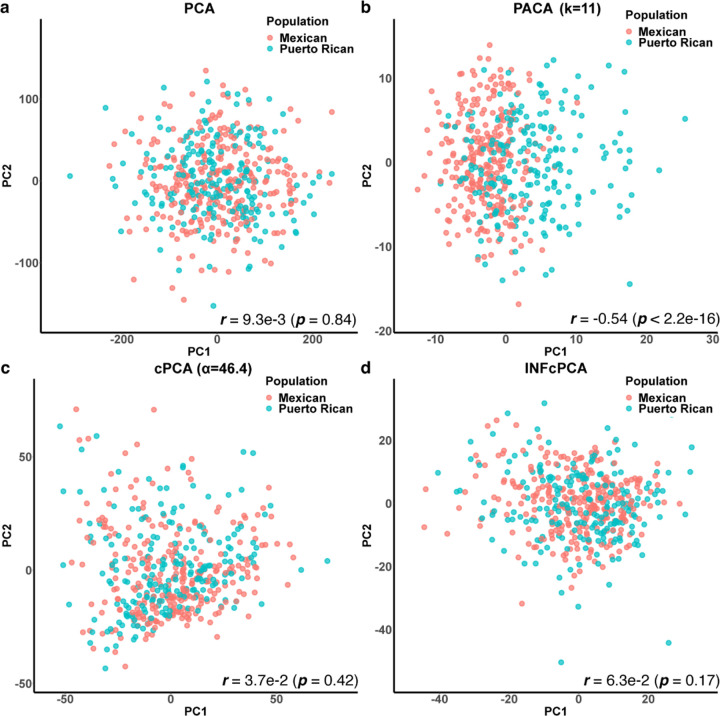
Capturing population heterogeneity in whole-blood DNA methylation data from admixed individuals. Presented are the first two components (denoted PC1 and PC2) found by (a) a standard PCA, (b) PACA, which accounted for an automatically estimated 11-dimensional structure of shared variation, (c) cPCA with the best performing regularization level among a range of values suggested by cPCA, and (d) cPCA with the regularization level set to *α* = *∞*.

The most dominant source of variation in DNA methylation from heterogeneous tissues such as blood is known to be cell-type composition [48], however it has been previously reported that a large number of methylation CpGs are strongly correlated with population structure [47, 63]. We therefore asked whether PACA can capture population stratification from heterogeneous methylation samples that were previously collected from individuals of Mexican and Puerto Rican descent (n=481) [48]. To that end, we applied PACA and cPCA to this target dataset while contrasting is with a background dataset of whole-blood methylation samples collected from European individuals (n=651) [64]. As expected, PACA achieved the best performance at capturing the target stratification in the data (Figure 3). On the other hand, cPCA could not capture the target variation either when considering the best performing regularization among a range of possible values of *α* suggested by cPCA (Figure 3c) or by setting *α* = ∞ (Figure 3d).

### 3.4 Identifying subphenotypic signal that reflects genetic heterogeneity under violation of the orthogonality assumption

Our simulation study suggests that PACA is more powerful than the alternatives in cases where the orthogonality assumption is not met, as long as the correlation between the subphenotypic signal and the shared sources of variation is weak or moderate (Supplementary Figure S2). In order to evaluate this result in real data, we applied PACA to a mixture of gene expression profiles from the PsychENCODE data [61, 62]. We pooled together schizophrenia (n=472) and bipolar (n=172) cases, the two most prevalent disorders in the data, as the “cases” group, thus, emulating a single disorder with two subtypes. For the background group, we considered all controls in the data (n=644). Prior to the analysis, we confirmed that the orthogonality assumption is violated: the simulated subtype was indeed correlated with the RNA sequencing technique used (library preparation) for the sample (poly(A) enrichment or ribosomal RNA depletion; r=0.12, p-value=0.003).

A simple application of PCA showed that the first PC of the cases group is correlated with the subtype assignment (r=−0.26, p-value=1.5e-11), however, this signal was driven by the correlation of the PC with the library preparation (r=0.70, p-value<2.2e-16). In fact, we found that the first two PCs of the data perfectly separates the samples by the library preparation type (Supplementary Figure S4). We therefore next asked whether applying PACA and cPCA using the controls group as a background data can capture subphenotypic signal that cannot be explained by the first two PCs of the data. While the first contrastive PC of cPCA did not capture substantial subphenotypic signal in that case (p-value=0.056 for the *α* resulting in highest correlation with the subtype; linear regression), the top PACA component of PACA did identify subtype signal when accounting for the first two PCs of the data (p-value= 2.6e-07; linear regression).

One way to evaluate whether the subphenotypic signal captured by PACA is meaningful (rather than merely reflecting other confounders) is by asking whether the subphenotypic signal captured by PACA is associated with genetic heterogeneity. A correspondence between the PACA component and changes in allele frequencies will provide strong indication for a real signal. In order to answer this question, we applied Subtest [65], a statistical method for the identification of genetic heterogeneity within phenotypically-defined subgroups (Supplementary Methods). Specifically, we applied Subtest on the genotypes of the PsychENCODE samples and provided it with the putative subphenotypic signal we learned using PACA. Indeed, Subtest confirmed that PACA captured an axis of subphenotypic variation that is indicative of differential genetics architecture among the cases (p-value <1.4e-6).

### 3.5 Capturing phenotypic heterogeneity in genetic data

Common genetic variation and specifically SNPs are notorious for their small effect sizes in complex disease [66, 67]. This makes the task of finding subtypes solely based on such genetic data particularly hard. We therefore evaluated the potential utility of PACA in identifying phenotypic heterogeneity from SNPs. To that end, we mixed genotype arrays of coronary artery disease (CAD) cases (n=2,500) and rheumatoid arthritis (RA) cases (n=2,500), which we pooled together from the UK Biobank data [68] into one group. Owing to the large number of genetic variants in typical genotype data, we worked under a relaxed assumption that prior work identified large sets of genetic variants that are likely to be associated with the phenotypic subtypes. Specifically, in our analysis, we considered the combination of a set of 6,000 random SNPs, the top 2,000 most associated SNPs with CAD, and the top 2,000 most associated SNPs with RA based on standard case-control GWAS (a total of 10,000 SNPs; Supplementary Methods). We applied PACA to this mixed group of cases while contrasting it with a group of healthy control individuals from the UK Biobank (n=5,000), and we found that the top PACA component is enriched for correlation with CAD- and RA-associated SNPs that represent a subphenotypic genetic heterogeneity in this case (Supplementary Figure S5a).

Finally, we applied Subtest to the data, which further confirmed that the top PACA component defines a spectrum of phenotypic heterogeneity that presents differential genetic architecture between the CAD and RA cases (p-value <4.201e-40). In total, we identify 211 SNPs (Subtest cFDR < 0.0434; Supplementary Methods) as likely to contribute to the difference in genetic basis of CAD and RA along the top PACA component (Supplementary Figure S5b).

## 4 Discussion

PACA is a contrastive learning algorithm for targeting weak sources of variation of interest from target data by leveraging background data that includes the same sources of variation that exist in the target data except for the variation of interest which is specific to the target data. Through an extensive simulation study and analysis of three genomic modalities, we demonstrated that PACA is calibrated and well-powered for identifying phenotypic heterogeneity. The power and robustness of our approach stems from the strategy of accounting for shared sources of variation across the target and background data. In particular, PACA aims at adjusting for the minimal number of shared axes of variation in the data that it is sufficient to remove in order to reveal variation that is specific to the target data. This strategy allows PACA to avoid falsely tagging confounders as subphenotypic signals and it subverts the need for a contrastive hyperparameter.

The robustness and calibration of PACA does come at a cost: our assumption that the subphenotypic signal is orthogonal to the confounders in the data may be violated in reality. A violation may occur either due to the nature of the subphenotypic signal or due to study-specific artefacts, such as case/control misclassification (i.e., noisy/incorrect labeling of controls as cases or vice versa). Not meeting this assumption may lead to power loss, however, we observe that PACA still supersedes the alternatives in scenarios of weak to moderate violation of the orthogonality assumption and comparable in cases of severe violation (Supplementary Figure S2). Furthermore, since PACA learns the directions of the shared sources of variation across *X*, *Y*, it can in principle be robust to imbalances in confounders between *X* and *Y*. However, much like any unsupervised method, PACA is expected to be sensitive to scenarios in which there are severe confounder imbalances or in cases where there are confounders that affect only *X* and not *Y*. If both *X* and *Y* were collected as part of the same study and under a proper randomization, such confounders in principle should not affect the data. Yet, in the event that such severe confounders are present in the data, they should be properly addressed. For example, in the case of multi-site data, if certain sites collected only cases or only controls then one should consider excluding the data from those sites prior to applying PACA. Otherwise, confounding effects due to site-specific variation will affect the ability of PACA to accurately capture the shared variation between cases and controls and the true cases-specific subphenotypic variation.

Finally, a straightforward implementation of the CCA backbone used by PACA restricts the number of samples to be lower than the number of features. That is, PACA is restricted to a “large-*p* small-*n*” regime (i.e., the number of features, *p*, is larger than the number of samples *n*). Yet, a desired property of a computational method is scalability and applicability to large sample sizes. In order to address this technical limitation of CCA, we developed a randomized version of PACA (rPACA), which allows us to apply PACA in regimes where *p* << *n* (Supplementary Methods).

## Supplementary Material

Supplement 1
