# Supplementary material for: Phenotypic subtyping via contrastive learning
Source: bioRxiv. 2023 Jan 6:2023.01.05.522921. Preprint. [Version 1] doi: 10.1101/2023.01.05.522921 (PMC9881932; doi:10.1101/2023.01.05.522921)
Supplement: Supplement 1 [file NIHPP2023.01.05.522921v1-supplement-1.pdf]

bioRxiv preprint doi: https://doi.org/10.1101/2023.01.05.522921; this version posted January 6, 2023. The copyright holder for this preprint (which was not certified by peer review) is the author/funder, who has granted bioRxiv a license to display the preprint in perpetuity. It is made available under aCC-BY 4.0 International license.

Supplementary Materials for:

Phenotypic subtyping via contrastive learning

Aditya Gorla, Sriram Sankararaman, Esteban Burchard, Jonathan Flint, Noah Zaitlen, and Elior Rahmani

bioRxiv preprint doi: https://doi.org/10.1101/2023.01.05.522921; this version posted January 6, 2023. The copyright holder for this preprint (which was not certified by peer review) is the author/funder, who has granted bioRxiv a license to display the preprint in perpetuity. It is made available under aCC-BY 4.0 International license.

Part I

Supplementary Figures

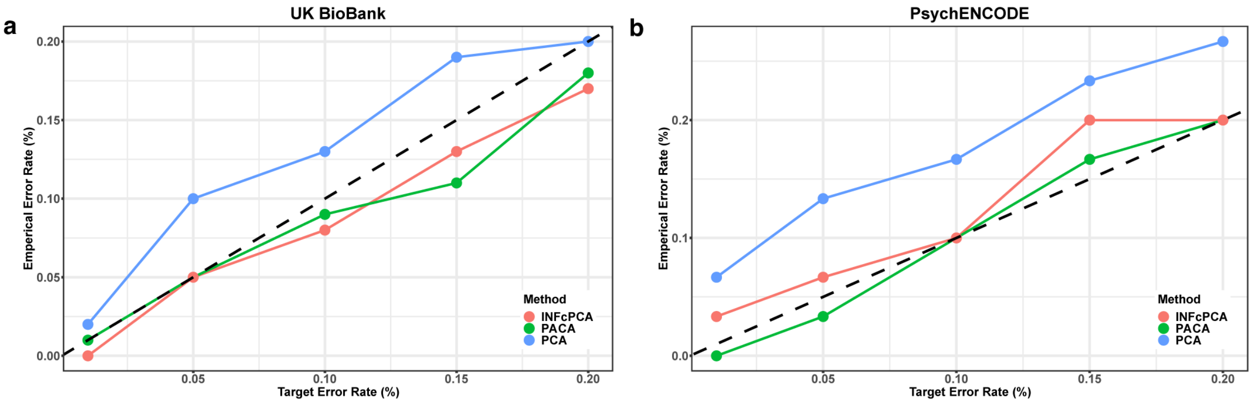

Figure S1: Evaluation of calibration under the null case of no subphenotypic variation. (a) The type I error calibration of PACA, cPCA ($\alpha = \infty$; INFcPCA) and PCA on genotype data from non-diseased unrelated white British individuals from the UKBiobank (N=2,000 and a randomly selected subset of 10,000 SNPs) over 100 simulations. (b) The type I error calibration on gene expression data on control samples from PsychENCODE (N=1,000 and 25,774 genes) over 30 simulations. The X-axes represent the target type-I error and the Y-axes correspond to the observed type-I error at the target error rate. The dotted lines represent an ideal type-I calibration curve.

bioRxiv preprint doi: https://doi.org/10.1101/2023.01.05.522921; this version posted January 6, 2023. The copyright holder for this preprint (which was not certified by peer review) is the author/funder, who has granted bioRxiv a license to display the preprint in perpetuity. It is made available under aCC-BY 4.0 International license.

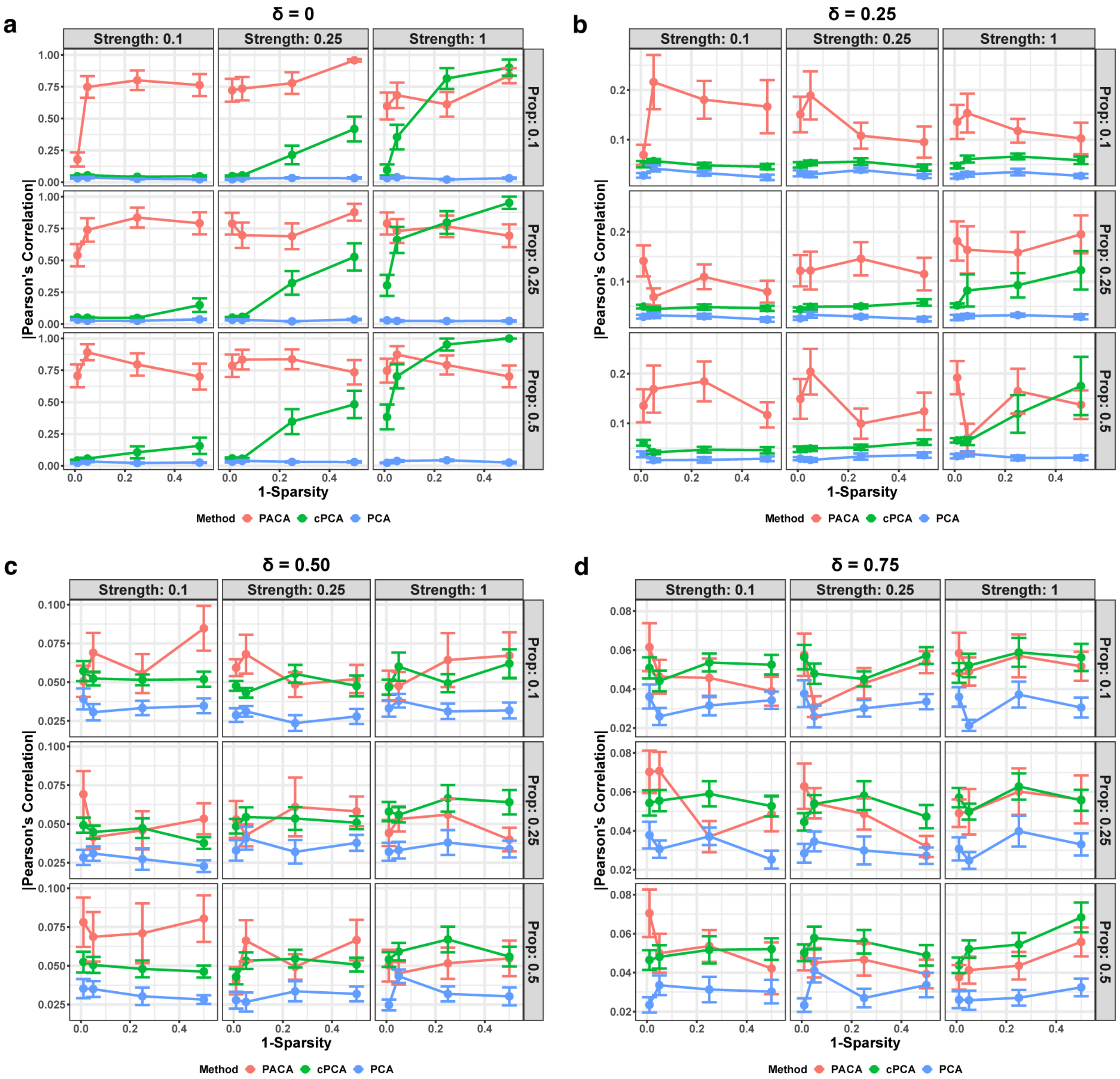

Figure S2: Evaluation of the power of different methods to capture subphenotypic signal under different levels of violation of the assumption of orthogonality between the cases-specific variability and the shared variability. For each of several levels of violation of the orthogonality assumption (denoted by δ), presented is the correlation of the outcome of the different methods with the subtype signal as a function of the signal strength, case subtype proportion (Prop), and signal sparsity (Supplementary Methods). δ corresponds to the ratio between the subtype variability in the controls and the subtype variability in the cases. Particularly, $\delta = 0$ corresponds to no subtype signal in the controls and $\delta = 1$ corresponds to the same level of subtype signal in cases and controls. Presented is the performance in terms of absolute Pearson correlation under the different configurations, averaged over 20 simulations with vertical bars representing standard errors. The results are based on simulated data with N=1,400, $k_0 = 300$, and 2,000 features.

bioRxiv preprint doi: https://doi.org/10.1101/2023.01.05.522921; this version posted January 6, 2023. The copyright holder for this preprint (which was not certified by peer review) is the author/funder, who has granted bioRxiv a license to display the preprint in perpetuity. It is made available under aCC-BY 4.0 International license.

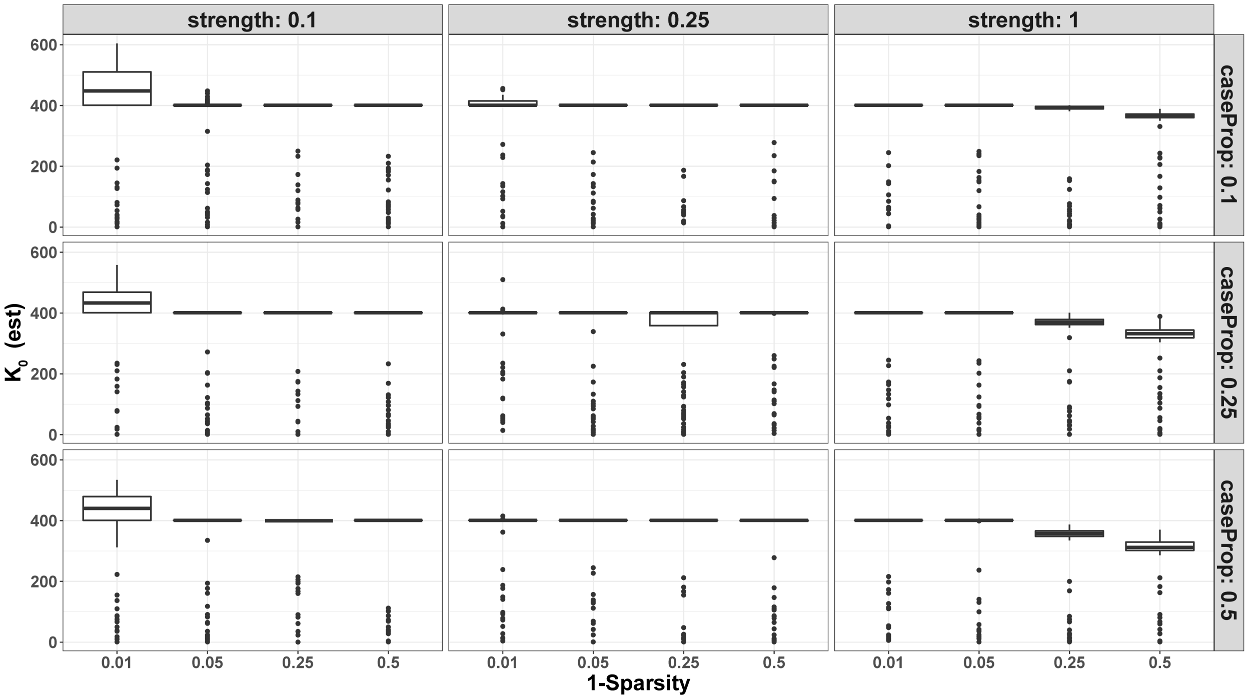

Figure S3: PACA estimates of the number of effective dimension of the shared variation between cases and controls (k) under simulations. The results are based on 100 simulations with $n = 2000$, $k_0 = 400$ and $m = 2000$. The columns are evaluations at fixed signal strength, the rows are evaluations at fixed case proportions, and the X-axes indicate the level of signal sparsity (lower values mean more sparse).

bioRxiv preprint doi: https://doi.org/10.1101/2023.01.05.522921; this version posted January 6, 2023. The copyright holder for this preprint (which was not certified by peer review) is the author/funder, who has granted bioRxiv a license to display the preprint in perpetuity. It is made available under aCC-BY 4.0 International license.

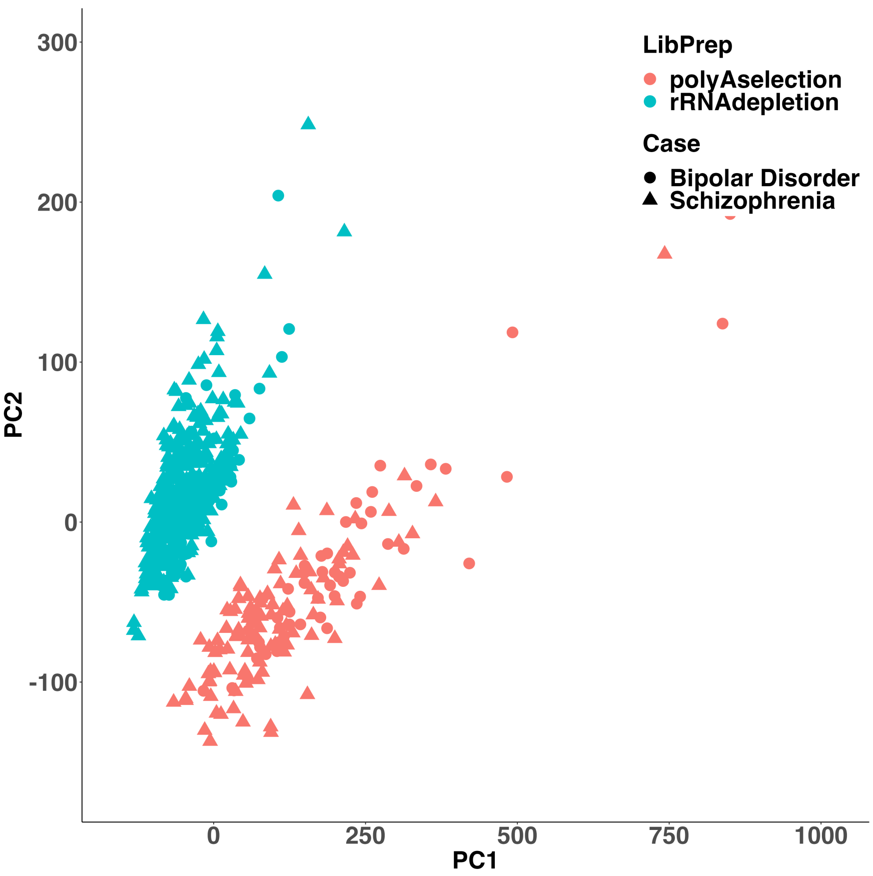

Figure S4: The top two principal components (PCs) of the pool of 472 schizophrenia cases and 172 bipolar disorder cases from the PsychENCODE data. The top two PCs perfectly separate the samples in the data based on the RNA Library preparation type, poly(A) selection (in blue) or rRNA depletion (in red).

bioRxiv preprint doi: https://doi.org/10.1101/2023.01.05.522921; this version posted January 6, 2023. The copyright holder for this preprint (which was not certified by peer review) is the author/funder, who has granted bioRxiv a license to display the preprint in perpetuity. It is made available under aCC-BY 4.0 International license.

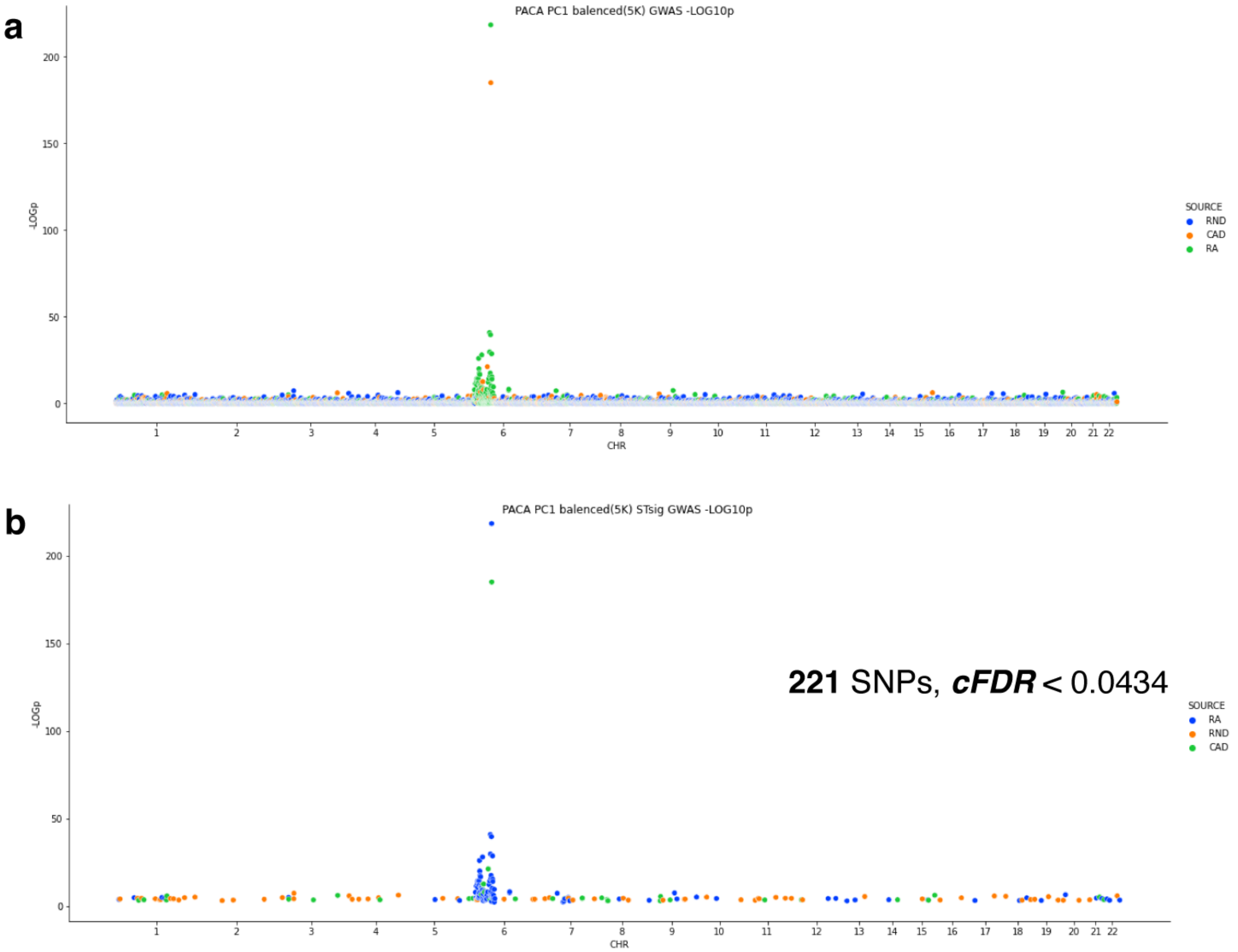

Figure S5: (a) Manhattan plot of a GWAS on pooled RA and CAD patients (N=10,000) with PACA PC1 as the phenotype. Results are shown for 10,000 SNPs, selected to be either RA-associated (green), CAD-associated (orange), or random (blue) SNPs. (b) Manhattan plot showing the 221 SNPs passing Subtest cFDR (<0.034). The 221 SNPs are either RA-associated (blue), CAD-associated (green), or random (orange) SNPs. The X-axes in the Manhattan plots correspond to chromosomal positions of the SNPs and the Y-axes reflect p-values on a -$\log_{10}$ scale.

bioRxiv preprint doi: https://doi.org/10.1101/2023.01.05.522921; this version posted January 6, 2023. The copyright holder for this preprint (which was not certified by peer review) is the author/funder, who has granted bioRxiv a license to display the preprint in perpetuity. It is made available under aCC-BY 4.0 International license.

Part II

Supplementary Methods

Table of Contents

S1 Datasets **8**
S1.1 HapMap 8
S1.2 Methylation Data 8
S1.3 UK BioBank 8
S1.4 PsychENCODE 8

S2 Simulations **9**

S3 cPCA evaluation **10**

S4 Evaluation Metrics **10**
S4.1 Power 10
S4.2 Null Calibration 10
S4.3 Subtest 10

S5 Implementation of PACA **11**

S6 Randomized PACA **11**

bioRxiv preprint doi: https://doi.org/10.1101/2023.01.05.522921; this version posted January 6, 2023. The copyright holder for this preprint (which was not certified by peer review) is the author/funder, who has granted bioRxiv a license to display the preprint in perpetuity. It is made available under aCC-BY 4.0 International license.

S1 Datasets

We used genotype data from the UK Biobank (UKBB) repsitory [1] and gene expression data from PsychENCODE [2, 3] for the null calibration experiment. We used both the aforementioned datasets and the genotype data of PsychENCODE for the validation analysis. In addition, we used the HapMap dataset [4] to illustrate null miscalibration.

S1.1 HapMap

We used genotypes from the HapMap project (release 23a, filtered) [4]. We considered a total of 120 samples (60 Yoruba in Ibadan, Nigeria (YRI) and 60 Northern/Western European Utah, U.S. (CEU) founders) and 1,872,641 SNPs for the analysis.

S1.2 Methylation Data

We used array DNA methylation datasets from the GALA II study with Latino population (n=573; GEO accession ID GSE77716) [5] and from the Hannum et al. study with European population (n=656; GEO accession ID GSE40279) [6]. We considered the top 50,000 most variable CpGs in the data. Prior to our analysis, we excluded outlier samples that demonstrated extreme values in the top two PCs of the data (values in PC1 or PC2 exceeding 3 standard errors), which resulted in n=558 and n=651 samples for the GALA II and Hannum datasets, respectively. Furthermore, the GALA II dataset includes 77 samples that are labeled as mixed Latino or other ethnicity; these were excluded from the final evaluation for which we only considered Mexican and Puerto Rican samples.

Mexican and Puerto Rican populations are known to be composed of European, Native American, and African populations. Therefore, the European samples in the Hannum data do not represent an ideal background dataset, given that information of European ancestry exists in the GALA II data. In our application of PACA we therefore employed a more relaxed assumption on the upper bound of the expected ratio between the variance of the subtype information in the Latino population compared with the variance of the subtype direction of information of the samples in the European population ($\delta = 4$).

S1.3 UK BioBank

We used a curated set of 291,273 unrelated white British individuals (459,792 SNPs) from the UK BioBank data (application No. 33127). This version of the data was used to calculate the first 10 PCs, which were uses as covariates in all GWAS runs. We used Field 20002 (Non-cancer illness, self reported; instance 1) from the phenotypic data to obtain disease type and status, and we classified individuals as healthy controls if they had no reported diseases in Field 20002; eventually, we randomly selected a subset of 5,000 controls.

For our Coronary Artery Disease (CAD) and Rheumatoid Arthritis (RA) analysis we also used Field 20002 in order to find 2,500 mutually exclusive cases of CAD and RA (randomly sampled from the complete groups of cases). Our analysis defines CAD patients as any individual who reported Angina or myocardial infraction. We considered a set of 10,000 SNPs, including 2,000 RA-associated, 2,000 CAD-associated and 6,000 random (null) SNPs. The 2,000 RA-associated and 2,000 CAD-associated SNPs were selected as the top mutually exclusive 2,000 SNPs for each disease using an LD Clumped ($\pm$ 100Kb window, LD < 0.1) list of the top SNPs from the Neal Lab summary statistics (v3; http://www.nealelab.is/uk-biobank/) present in our dataset. For the null SNPs, we randomly choose 6,000 SNPs from a LD pruned list (LD< 0.1) that had no overlap with the 2,011 and 9,696 most significant RA- and CAD-associated SNPs, respectively.

S1.4 PsychENCODE

We used genotypes, gene expression, and covariate information for 472 schizophrenia (SCZ), 172 bipolar disorder (BP), and 33 Alzheimer's disease (AD) patients, as well as for 644 controls. For the validation analysis, we only used SCZ, BP and controls. We used all 5,312,508 SNPs and all samples to learn the top 10 PCs which we used a covariates in all GWAS runs. We standardized all the raw counts of the 25,774 genes in the gene expression data, and we used the a set of 217,863 LD pruned SNPs (< 0.1) for the Subtest analysis.

bioRxiv preprint doi: https://doi.org/10.1101/2023.01.05.522921; this version posted January 6, 2023. The copyright holder for this preprint (which was not certified by peer review) is the author/funder, who has granted bioRxiv a license to display the preprint in perpetuity. It is made available under aCC-BY 4.0 International license.

S2 Simulations

We generate complete simulations as outlined in this section for the power analysis. We simulated data with two subtypes using the following model. Let $X_0 \in \mathbb{R}^{m\times n_0}, X_1 \in \mathbb{R}^{m\times n_1}$ be the data for controls and cases, respectively, we assumed:

$$X_0 = WZ_0 + E_0$$
$$X_1 = WZ_1 + W'Z_1' + E_1$$
$$W \in \mathbb{R}^{m\times k}, \quad W' \in \mathbb{R}^{m\times 2}$$
$$Z_0 \in \mathbb{R}^{k\times n_0}, \quad Z_1 \in \mathbb{R}^{k\times n_1}, \quad Z_1' \in \mathbb{R}^{2\times n_1}$$
$$E_0 \in \mathbb{R}^{m\times n_0}, \quad E_1 \in \mathbb{R}^{m\times n_1}$$

We simulated the the direction of the structure in the data, W, W', as follows:

$$W_j' \sim \pi_0\delta_{\mathbf{0}} + (1-\pi_0)N(0, \sigma^2\mathbf{I_m})$$
$$W_j \sim N(0, \sigma_j^2\mathbf{I_m})$$
$$\sigma_j^2 \sim \text{IG}(\alpha = 1.5, \beta = 1)$$

where $\pi_0 \in [0,1]$ reflects the signal sparsity. The signal sparsity controls the proportion of features with no effect (i.e., zero weights) in the k latent factors. $1-\pi_0$ can therefore be interpreted as the proportion of features affecting each of the k latent factors; for example, a fraction of 0.2 implies that a random combination of 20% of the m features define each of the k latent factors. We set $\sigma^2 = 3$ in all of our simulations, and in the null case we set $W' = \mathbf{0}$.

Next, we simulate the structure in the shared and unique structure. Let k be the number of shared dimensions of structure across cases and controls. For $k' \leq k$, let k' be the dim of the balanced shared variation, and $k-k'$ be the dim of the unbalanced shared variation. In the balanced axes of variation for $i \in \{1,\dots,k'\}$:

$$(Z_0)_i, (Z_1)_i \sim N(0, \sigma_i^2\mathbf{I_n})$$
$$\sigma_i^2 \sim \text{IG}(\alpha = 1.5, \beta = 1)$$

For the imbalanced axes of variation, when $k' \neq k$, $i \in \{k'+1, ..., k\}$:

$$(Z_0)_i \sim N(0, \sigma_i^2\mathbf{I_{n_0}})$$
$$(Z_1)_i \sim N(0, \tilde{\sigma}_i^2\mathbf{I_{n_1}})$$
$$\tilde{\sigma}_i^2, \sigma_i^2 \sim \text{IG}(\alpha = 1.5, \beta = 1)$$

We simulate the subtype structure (membership), Z_1', as one-hot encoded matrix assignment of individuals to the subtypes. We define "Prop" as the proportion of cases which belong to the first subtype. This is encoded by non-zero values in first column of Z_1'. "Prop" helps us analyze the performance of methods under different levels of imbalance between the prevalence of subtypes. We can alter the subtype signal "Strength" by altering the Z_1' encodings' non-zero values. "Strength" effectively scales the variance of the cases along the subspace which distinguishes the two subtypes. When defining a spectrum of phenotypic heterogeneity, instead of assigning individuals into subtypes, we sampled values from a standard random normal distribution.

We would also like to explore scenarios where the case specific subphenotypic signal is not exactly orthogonal to the structure in the controls. To explore this, we can also add some of the case specific structure to the controls $(\delta)^2 * W'Z_0'$. W' is the same direction of structure as in the cases, Z_0' encode random assignment of controls to one of the two subgroups, and $\delta \in [0,1]$ controls the ratio of the variance of the subphenotypic signal in cases and the variance of the subphenotypic signal in controls. Pulling all this together we have the following model:

$$X_0 = WZ_0 + (\delta)^2 * W'Z_0' + E_0$$

Finally, We simulate the noise terms from a normal distribution:

$$(E_0)_{ij} \sim N(0, \sigma_0^2)$$
$$(E_1)_{ij} \sim N(0, \sigma_1^2)$$

where $\sigma_0^2, \sigma_1^2 \in [0,1]$ controls the strength of the noise added to the controls and cases, respectively.

bioRxiv preprint doi: https://doi.org/10.1101/2023.01.05.522921; this version posted January 6, 2023. The copyright holder for this preprint (which was not certified by peer review) is the author/funder, who has granted bioRxiv a license to display the preprint in perpetuity. It is made available under aCC-BY 4.0 International license.

S3 cPCA evaluation

The optimal choice of the contrastive hyperparameter α is unclear without prior information. We instead choose to perform an overly optimistic evaluation of cPCA in a setting where we have the subtype information and can estimate estimate the optimal contrast value. To do so for every run, we let cPCA automatically evaluate at 10 different contrastive hyperparameter values and only select the hyperparameter yielding the highest correlation with the subtype signal. This represents a setting where the subtype information is know *a priori* and could be considered the near best-case performance for cPCA. That being said, if we force cPCA to return a single contrastive hyperparameter it always, in our experiments, returns a value of 0. In such cases we use a small offset to set $\alpha = 0.01$. As expect, the results seen at this setting were indistinguishable from PCA. In the PsychENCODE and methylation data analysis, we select the α which maximizes the correlation of cPC1 with the subtype of interest among the list of values suggested by cPCA. Finally, we also assess the results of cPCA at $\alpha = \infty$ (INFcPCA). In practice its not possible to actually set the contrastive hyperparameter to ∞, instead we set it to an extremely high value ($\alpha = 10^6$).

S4 Evaluation Metrics

S4.1 Power

For performance evaluation, we use the Pearson's correlation between a candidate subtype signal and the true subtype labels as a metric for power. To assess power, we use complete simulations instead of augmenting real data. There are several reasons we do this. Firstly, we want to assess methods under our model assumptions and its best to do this under complete simulations as we can control most properties of the data. Additionally, it is not trivial to add external structure to null genetic/gene expression data without fundamentally changing the nature of the data. Doing so could make it either trivial to detect the signal because it is distributionally distinct from the generative distribution of the base data or it could be stronger than any shared variation. Secondly, when using real data we do not have an *a priori* estimate of the number of shared dimensions between the case and controls, which makes it impossible to evaluate our k-selection procedure. Finally, our simulation framework allows us to control the presence/absence of imbalanced confounder to simulate more realistic data regimes and understand the impact of violating the orthogonality assumption. Such analysis is either not possible or quite hard when using real data since we have no control over the true undying shared structure.

S4.2 Null Calibration

We used a straight forward case/control label permutation approach to quantify the statistical significance of a tentative subtype signal at a given k, or α for cPCA. At a fixed k, we build a null distribution of variances of the top PC by running PACA on all the permuted datasets. Then use this empirical null distribution to quantify the statistical significance of the observed top PC's variance. This procedure should be able to reject the null when there is sufficiently strong variation unique to the cases. The exact same procedure is also used to quantify the statistical significance of the top PCA and cPCA components.

Note that we do not run analysis for the Null case using simulations because we are able to evaluate under more realistic null settings when we randomly split (real) healthy samples randomly into cases and controls. The real data we use for null calibration evaluation may contain variation that stem from unknown and possibility imbalanced sources. These factors make this evaluation procedure a better proxy for the settings of real applications. We do acknowledge that the randomization of samples may not sufficiently suppress unknown variation that could be tagged as a possible subtype. This, however, simply means that our real data calibration results are conservative relative to evaluation with simulated data under a perfect/complete null, which we rarely observe in real data.

S4.3 Subtest

Finally, we use Subtest [7], to check if a particular axis of variation defines an axis of genetically different architecture. Subtest is a statistical test to determine the existence of phenotypic subgroups in genetics. It does so by modeling the distribution of all SNP association statistics and fits a mixture of Gaussians. Explicitly, it tries to fits SNPs to 3 Bivariate Gaussian : 1) a distribution of SNPs associated with neither the disease or proposed subtype, 2) a distribution of SNPs only significantly associated with the disease

bioRxiv preprint doi: https://doi.org/10.1101/2023.01.05.522921; this version posted January 6, 2023. The copyright holder for this preprint (which was not certified by peer review) is the author/funder, who has granted bioRxiv a license to display the preprint in perpetuity. It is made available under aCC-BY 4.0 International license.

and not the subtype and 3) a distribution of SNPs which are either (i) associated only with the subtype or (ii) significantly associated with both the disease and proposed subtypes. Subtest fits Null model with a mixtures of model 1), 2) and 3)(i) and the alternate model with 1), 2) and 3)(ii), the difference of these model likelihoods provides a (pseudo) LogLikelihoods Ratio (pLR). We can generate a null distribution by permuting the subtype values, generating the null test statistics and fitting the Subtest to this data, which generates the null distribution for the pLR, which we can now use to derive a p-value quantify the the significance the proposed subtypes.

Fundamentally, Subtest assumes that if valid subtypes exist, they should have some non-negligible proportion of SNPs which are associated with the subtypes and the primary disease and it tries to find the existence of such subtypes. We don't believe that this models encompasses the full space of all possible subtypes, nonetheless this a well-motivated and well-defined set space for validating putative subtypes. In our application, we are using genetics to validate proposed subtypes uncovered by PACA in gene expression or genetic data. Therefore, we can use Subtest to test whether PACA PC1 learns a valid axis of variation that defines a genetically differential subtype. Please also note that Subtest also provides a way to test for subtype differences in each SNP using a Bayesian conditional False Discovery Rate (cFDR), by conditioned the subgroup test statistic on the primary phenotype test statistic for each SNP. We can use this procedure to select for SNPs which tentatively discriminate the proposed subtypes. We only provide a very brief introduction to Subtest here, please refer to [7] for a more detailed explanation on the topic.

S5 Implementation of PACA

PACA is mainly implemented in R, however, for the core CCA worker function we used a recently developed highly efficient cpp implementation [8].

All analysis, execution and visualization were performed in a series of R and Python (for cPCA and PCA) scripts. PACA will be available as a R package which will be open sourced and make public upon publication.

S6 Randomized PACA

The CCA step in PACA imposes the limitation $m \geq \max(n_0, n_1)$. In practice, there may be datasets in which this condition does not hold. In such cases, we can apply PACA on a subset of the samples, however, this is clearly sub-optimal to exclude data. In order to address this limitation, we introduce randomized PACA (rPACA), a randomized variant of PACA, which can operate under the setup $m < \max(n_0, n_1)$ and provide cases-specific variation for all n_1 samples in X. rPACA is based on the idea that the full sample covariance matrix of the cases (after adjustment for shared sources of variation) can be approximated by learning projections from multiple subsets of the samples in the data. Finding the eigenvector that corresponds to the top eigenvalue of the estimated sample covariance matrix will then allow us to describe the most dominant variation in X while accounting for the sources of variation that are shared with Y. Below, we provide a brief description of the rPACA algorithm; see Supplementary Algorithm 2 for the complete details.

Briefly, rPACA begins by randomly splitting the data into two sets, each with a subset of the cases and a subset of the controls. rPACA uses one of the sets to learn the shared axes of variation between the cases and controls in the set, which are then used to adjust cases in both sets of the data. We then obtain the top q PCs of both sets of cases and stack them into a single matrix; of note, we match the variances of the projections across the two subsets of cases, along each of the q PCs, which alleviates possible variance shrinkage. Repeating this procedure multiple times using multiple splits of the data and combining the PCs across the multiple iterations allows us to use them to approximate the sample-covariance matrix in X. So we believe we should be able to randomly sub-sample the case/control data to approximate the full corrected case covariance matrix which can be used to find the top case variation specific PC.

bioRxiv preprint doi: https://doi.org/10.1101/2023.01.05.522921; this version posted January 6, 2023. The copyright holder for this preprint (which was not certified by peer review) is the author/funder, who has granted bioRxiv a license to display the preprint in perpetuity. It is made available under aCC-BY 4.0 International license.

Algorithm S1 Randomized PACA (rPACA)

Input: $X \in \mathbb{R}^{m \times n_1}$, $Y \in \mathbb{R}^{m \times n_0}$, t number of iterations, $s < \min\{m, n_1, n_0\}$ batch size, k dimension of shared variation to remove.

$X'_{ij} \leftarrow X_{ij} - \frac{1}{m}\sum_{k=1}^{m} X_{kj},\ Y'_{ij} \leftarrow Y_{ij} - \frac{1}{m}\sum_{k=1}^{m} Y_{kj}$ ▷ Mean centering the matrices

$P \leftarrow \mathbf{0}$

for $n \leftarrow 1, ..., t$ **do**

  $X''_1, X''_2 \leftarrow \Pi_s(X'),\ Y''_1, Y''_2 \leftarrow \Pi_s(Y')$ ▷ $\Pi_s(.)$: randomly sample s as train(1) and rest as test(2) set

  for $r \leftarrow 1, ..., k$ **do**

$$\begin{aligned} \hat{a}_r, \hat{b}_r \quad &= \quad \underset{a_r \in \mathbb{R}^B, b_r \in \mathbb{R}^B}{\operatorname{argmax}} \ a_r^\top X_1''^\top Y_1'' b_r \\ \text{s.t.} \quad & \quad \|X''_1 a_r\|_2 = 1, \|Y''_1 b_r\|_2 = 1 \\ & \quad \forall 1 \le h < r : X''_1 a_h \perp X''_1 a_r, Y''_1 b_h \perp Y''_1 b_r \end{aligned}$$

  $\hat{A} \leftarrow [\hat{a}_1, ..., \hat{a}_k]$

  $\hat{U}^0 \leftarrow X''_1 \hat{A}$

  $\tilde{X}_1 \leftarrow X''_1 - \hat{U}^0 (\hat{U}^0)^\top X''_1$

  $\hat{V}_{X_1}, \hat{Z}_{X_1} \leftarrow \text{PCA}(\tilde{X}_1{}^\top)$

  $\tilde{X}_2 \leftarrow X''_2 - \hat{U}^0 (\hat{U}^0)^\top X''_2$

  $\hat{V}_{X_2}, \hat{Z}_{X_2} \leftarrow \text{PCA}(\tilde{X}_2{}^\top)$

  $P' \leftarrow \begin{bmatrix} \hat{V}_{X_1} \\ \hat{V}_{X_2} \end{bmatrix}$ ▷ Stack projections to match order of X

  $P \leftarrow [P\ P']$ ▷ Hstack projection from each iteration

$C = PP^\top$

$\hat{V}_X \leftarrow \underset{||\mathbf{v}||_2 = 1}{\operatorname{argmax}} ||C\mathbf{v}||_2$ ▷ First eigenvector of the covariance matrix

Output: $\hat{V}_X$

bioRxiv preprint doi: https://doi.org/10.1101/2023.01.05.522921; this version posted January 6, 2023. The copyright holder for this preprint (which was not certified by peer review) is the author/funder, who has granted bioRxiv a license to display the preprint in perpetuity. It is made available under aCC-BY 4.0 International license.

References

[1] Clare Bycroft et al. "The UK Biobank Resource With Deep Phenotyping and genomic data". In: *Nature* 562.7726 (2018), 203–209.

[2] Michael J. Gandal et al. "Transcriptome-wide isoform-level dysregulation in ASD, schizophrenia, and bipolar disorder". In: *Science* 362.6420 (2018).

[3] Daifeng Wang et al. "Comprehensive functional genomic resource and integrative model for the human brain". In: *Science* 362.6420 (2018).

[4] International HapMap Consortium et al. "The international HapMap project". In: *Nature* 426.6968 (2003), p. 789.

[5] Elior Rahmani et al. "Sparse PCA corrects for cell type heterogeneity in epigenome-wide association studies". In: *Nature methods* 13.5 (2016), p. 443.

[6] Gregory Hannum et al. "Genome-wide methylation profiles reveal quantitative views of human aging rates". In: *Molecular cell* 49.2 (2013), pp. 359–367.

[7] James Liley, John A Todd, and Chris Wallace. "A method for identifying genetic heterogeneity within phenotypically defined disease subgroups". In: *Nature genetics* 49.2 (2017), pp. 310–316.

[8] Mike Thompson et al. "Confined: Distinguishing biological from technical sources of variation by leveraging multiple methylation datasets". In: *Genome Biology* 20.1 (2019).
